# Effects of short indels on protein structure and function in human genomes

**DOI:** 10.1038/s41598-017-09287-x

**Published:** 2017-08-24

**Authors:** Maoxuan Lin, Sarah Whitmire, Jing Chen, Alvin Farrel, Xinghua Shi, Jun-tao Guo

**Affiliations:** Department of Bioinformatics and Genomics, The University of North Carolina at Charlotte, Charlotte, NC 28223 USA

## Abstract

Insertions and deletions (indels) represent the second most common type of genetic variations in human genomes. Indels can be deleterious and contribute to disease susceptibility as recent genome sequencing projects revealed a large number of indels in various cancer types. In this study, we investigated the possible effects of small coding indels on protein structure and function, and the baseline characteristics of indels in 2504 individuals of 26 populations from the 1000 Genomes Project. We found that each population has a distinct pattern in genes with small indels. Frameshift (FS) indels are enriched in olfactory receptor activity while non-frameshift (NFS) indels are enriched in transcription-related proteins. Structural analysis of NFS indels revealed that they predominantly adopt coil or disordered conformations, especially in proteins with transcription-related NFS indels. These results suggest that the annotated coding indels from the 1000 Genomes Project, while contributing to genetic variations and phenotypic diversity, generally do not affect the core protein structures and have no deleterious effect on essential biological processes. In addition, we found that a number of reference genome annotations might need to be updated due to the high prevalence of annotated homozygous indels in the general population.

## Introduction

Insertions and deletions (indels) are additions or deletions of one or more nucleotides in DNA sequence. Indels are highly abundant in human genomes, second only to single nucleotide polymorphisms (SNP), and make up 15–21% of human polymorphisms^1^. Indels in coding regions can result in two different types of variants, frameshift (FS) and non-frameshift (NFS). NFS indels consist of a multiple of three base pairs, introducing an insertion or deletion of one or more amino acids while keeping the rest of the protein sequence unchanged. In contrast, FS indels change the reading frame starting from the site of insertion/deletion, which can produce different protein sequences or lead to premature termination and the mRNA can be subjected to a surveillance pathway called non-sense-mediated mRNA decay (NMD)^2^. A rate of 2.94 indels (1–20 bp) and 0.16 structural variants (>20 bp) per generation was estimated based on whole genome sequencing of 250 families^3^. While regarded as an alternative of natural genetic variation to SNP, previous studies have demonstrated the role of indels in the development of a number of Mendelian diseases^4–6^. For example, cystic fibrosis, with an incidence rate of 1 in 3500 in North America, is caused by a three base-pair deletion within the *CFTR* (Cystic Fibrosis Transmembrane Conductance Regulator) gene^7, 8^. Indels have also been implicated in diseases including acute myeloid leukemia^9, 10^ and other types of cancer^11^.

With the advancement of sequencing techniques and cost reduction, a large number of personal genomes, both from healthy individuals and cancer patients have been sequenced, which sped up the process of building a comprehensive catalog of indel variants^1, 12–22^. For example, the 1000 Genomes Project, the largest public catalogue of human variation and genotype data, has recently completed its final phase in 2015^23, 24^. The project sequenced 2,504 individual genomes representing 26 diverse populations in Africa (AFR), the Americas (AMR), East Asia (EAS), Europe (EUR), and South Asia (SAS). The landscape of natural genetic variations and somatic mutations, including indels, has been investigated in an attempt to discover deleterious mutations^19–21, 25–27^. Several machine-learning methods have been developed to predict the phenotypic effect of both FS^28–30^ and NFS indels^5, 30–32^. The disease-causing indels are generally derived from the Human Gene Mutation Database (HGMD), while the neutral indels are from the 1000 Genomes Project or curated from protein sequence databases. The structural effects of small NFS indels have also been investigated using protein isoform structures or highly homologous protein structures in Protein Data Bank (PDB)^33–35^. Results show that protein structures can tolerate small natural indels as the majority of indel residues are exposed to the solvent and about one-third of residues are in disordered state^35^.

While efforts have been devoted to predictions of potential pathogenicity of small indels, there are no comprehensive studies of the effect of short coding indels on protein structure and function in a large number of human genomes. In this study, we focused on the analysis of short coding indels (<50 bp) in the 1000 Genomes Project to explore the role of these genetic variations in protein structure (for NFS indels) and function (for both FS and NFS indels), which can serve as background characteristics for studying disease-causing indels in various diseases. In addition, we identified a number of genes with homozygous FS and NFS indels that have very high frequency among the diverse populations, which may serve as basis for future reference genome updates.

## Results and Discussion

### Distribution of short coding indels

There are a total of 769,743 short coding indels in the 2,504 human genomes, where raw indels were first called based on the numbers of reads supporting reference and alternative alleles and the genotypes of these indels were further refined by considering SNPs genotypes and haplotype structure^23, 24^. While some coding indels are rare variants, 209 homozygous indels (72 NFS and 137 FS) were found in over 50% of the 2,504 individuals (Supplementary Table S1). Among them, 1 NFS and 61 FS homozygous indels appear in all 2,504 genomes (Supplementary Tables S2 and S3). These high frequency homozygous indels should be a point of interest for human reference genome updates as suggested in structural variants studies^23^. The number of unique indels is 7,137 (if the same indel occurs in multiple genomes, it only counts as one unique indel). There are slightly more FS indels (3,775) than NFS indels (3,362). About 37% (1240/3362) of the NFS indels and 32.5% (1226/3775) of the FS indels have more than 1% of allele frequency.

**Figure 1:**
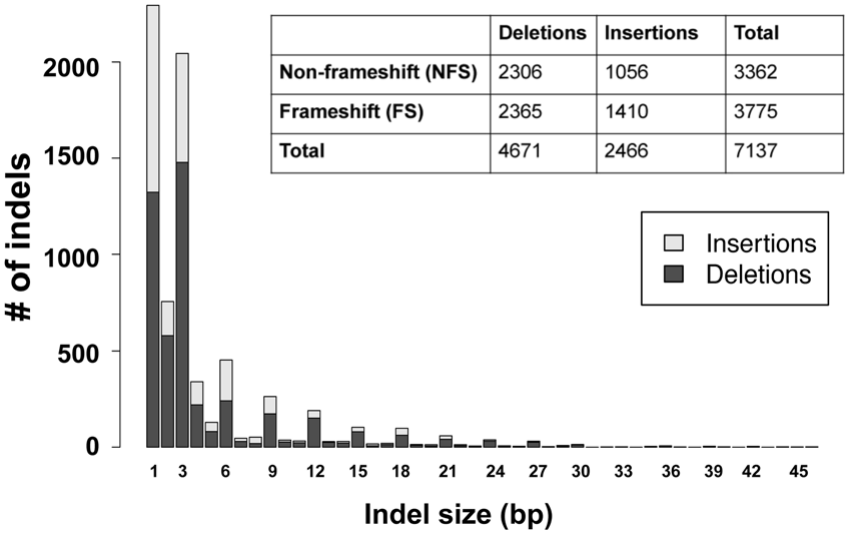
Indel size distribution.

There are about twice as many deletion indels (4,671) than the insertion indels (2,466) (Fig. 1). Short coding indels are highly enriched. Insertions and deletions of one to three nucleotides represent about 70% of all unique coding indels. Except for the single nucleotide indel, which has the highest occurrence, there are more NFS indels (multiple of three nucleotides) than FS indels in each of the three nucleotides window (Fig. 1). Since two out of three codon positions result in FS indels, the number of FS indels is smaller than expected, which is not surprising as FS indels are considered more deleterious, and mutants with such indels are more likely to be removed from population through purifying selection^36, 37^. The actual number of FS indels could be even smaller after updates of the human reference genome in the future since a number of homozygous FS indels appear in every individual genome (Supplementary Table S3). In addition, in some cases a second FS indel may rescue a potential deleterious variant of the first FS indel by correcting the open reading frame. For example, a 2 bp insertion at position 74,836,315 on one individual’s *ARID3B* gene (ENSG00000179361) can be rescued by a 1 bp insertion at the next position 74,836,316. However, if the two FS positions are so far away, it makes it a different protein sequence between the two variant sites. Gene *CLTCL1* (ENSG00000070371) on one genome is such an example. It has a 1 bp insertion at position 19,189,003 and a 10 bp deletion at position 19,170,999. Even though the combination of these two FS indels results in a 9 bp deletion, a relatively larger piece of the protein sequence involving several exons is changed. A list of genes with at least two FS indels on one individual’s same gene is shown in Supplementary Table S4. Not only can an FS indel introduce premature stop codon, NFS can also introduce a premature stop codon, we found a total of eight such unique cases (Supplementary Table S5).

**Figure 2:**
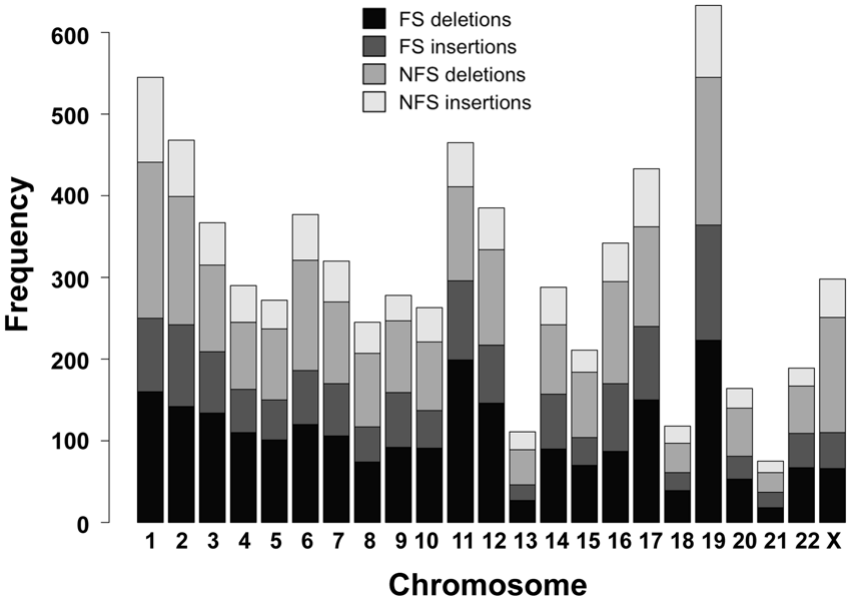
Number of unique indels on each chromosome.

**Figure 3:**
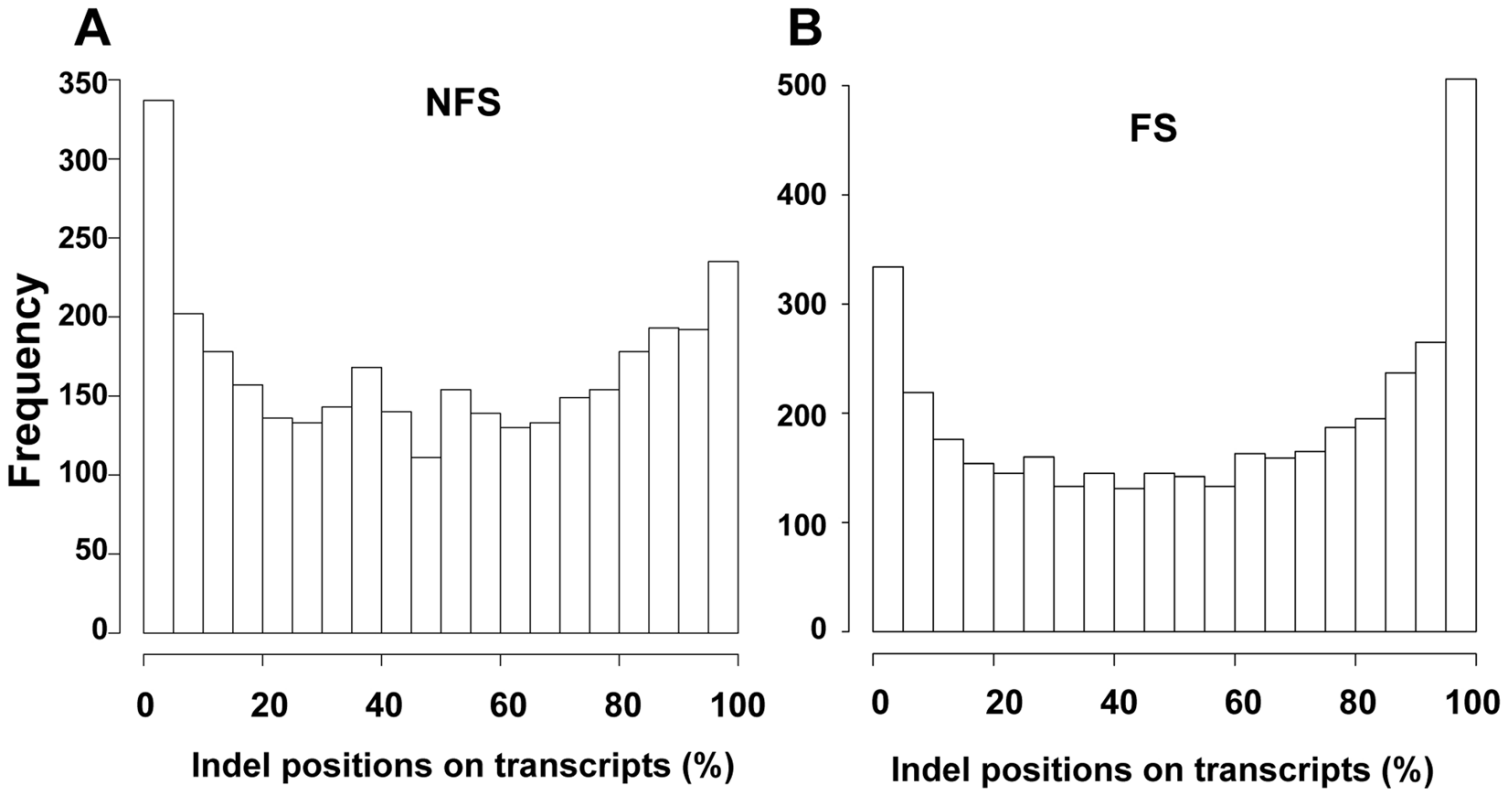
Relative positions of NFS (**A**) and FS (**B**) indels on proteins.

The distribution of the unique indels on each chromosome is shown in Fig. 2. While the chromosomes are generally numbered from the largest to the smallest, the protein coding genes are unevenly distributed across the chromosomes. For example, chromosome 19, one of the smallest chromosomes, has the highest gene density of all human chromosomes^38, 39^. The next highest gene dense chromosomes are 17, 22, 16, and 11, while the lowest density chromosomes are 13 and 18^39^. Therefore the number of unique indels on each chromosome is closely related to its number of protein coding genes (Fig. 2). Coding indels are enriched in N- or C-terminal regions (Fig. 3). It is not surprising to observe that there are more N-terminal indels in the NFS cases and more C-terminal indels in the FS cases. In both situations, a majority of the protein sequences are not changed and indels should have minimal effects on the structure and function of affected proteins.

**Figure 4:**
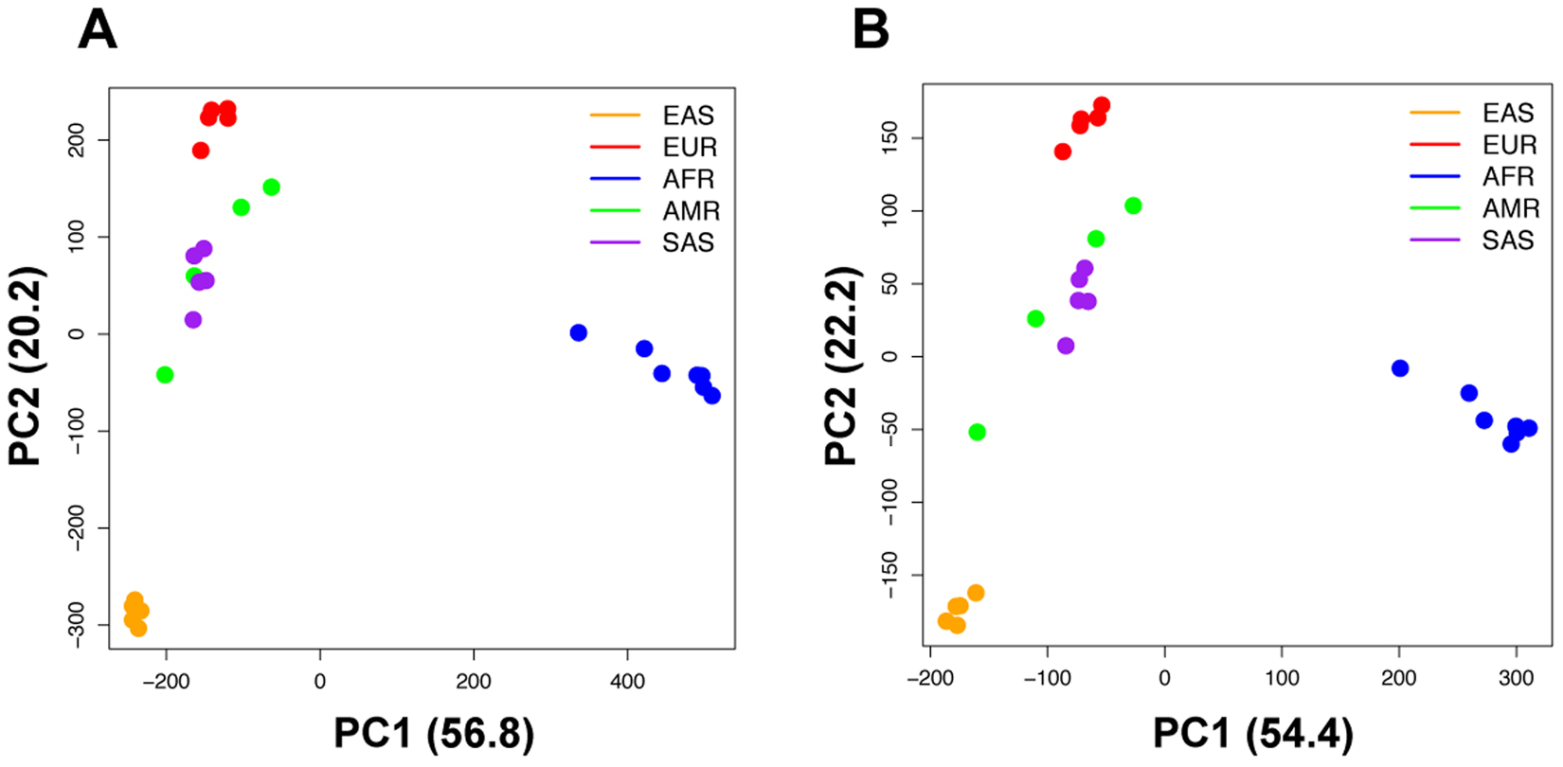
PCA analysis of indel patterns in 26 populations. (**A**) All indels; (**B**) Homozygous indels only

Principal component analyses (PCA) of indel patterns revealed the clustering of 26 populations into their respective five super populations (Fig. 4). There are no clear differences between the results from all indels (Fig. 4A) and homozygous indels (Fig. 4B). The first two principal components PC1 and PC2 explain about 77% of the variations of the indel patterns. In both cases, AFR can be clearly separated from other super populations by the first principal component, and the second principal component further separates EAS and EUR from the other two ancestries: SAS and AMR. There is some overlap between AMR and SAS based on the first two principal components. Our indel PCA results are consistent with the broad patterns from structural variants (SV, defined as DNA variants of more than 50 bp) PCA analyses^23^. The data suggest that each super population has its own distinct patterns of indels, which may potentially contribute to the phenotypic differences among the populations. For example, an FS indel on *GPR142* (ENSG0000257008) was only found in AFR super populations and another indel on *LGR6* (ENSG0000133067) has different frequencies in AMR, AFR and SAS with zero occurrences in EUR and EAS. Recent report on global reference for human genetic variants revealed similar results^24^. About 762,000 rare variants (<0.5% in full population) were found frequently in at least one population (>5%) and populations with higher numbers of variants were geographically separated. This is especially true for the AFR populations^24^.

### Functional enrichment analysis

**Figure 5:**
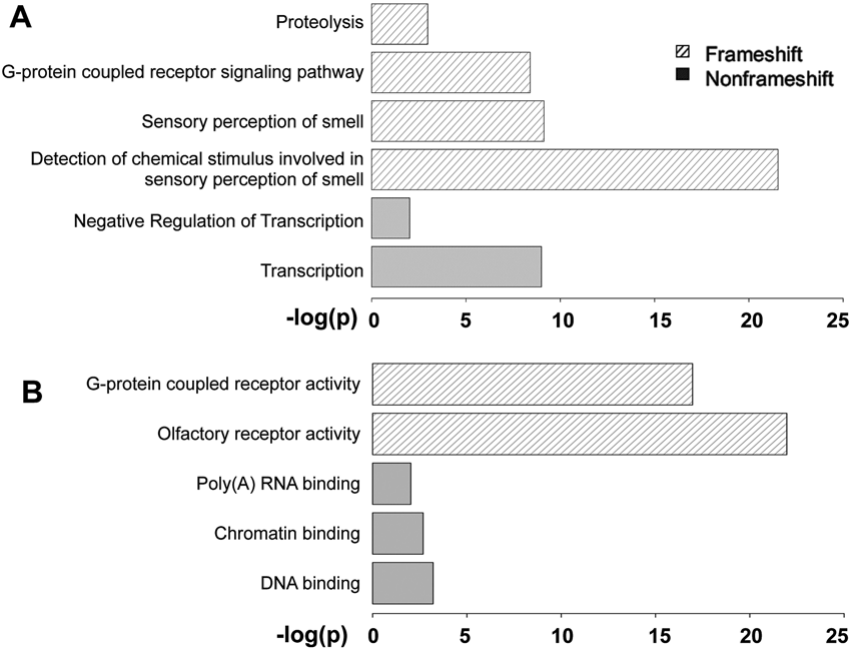
Gene enrichment analysis of genes with NFS or FS indels. (**A**) Significantly enriched categories in terms of Biological Process; (**B**) Significantly enriched categories in terms of Molecular Function.

To investigate possible associations between short coding indels and functional categories of the affected proteins, we applied DAVID v6.8, the “Database for Annotation, Visualization and Integrated Discovery” and performed functional enrichment analysis^40^. The categories are analyzed based on Gene Ontology (GO)’s Biological Process and Molecular Function annotations respectively and the significantly enriched categories were selected using an FDR threshold of 0.05^41^. We observed different enrichment patterns between genes with FS and NFS indels (Fig. 5). In terms of biological process, the top three significantly enriched categories in FS related genes are all olfactory-related: detection of chemical stimulus-smell, sensory perception of smell, and G-protein coupled receptor signaling pathway (Fig. 5A). In NFS cases, transcription-related biological processes are highly enriched (Fig. 5A). Results from molecular function enrichment analysis are consistent with corresponding biological process data (Fig. 5B).

Olfactory receptor activity and G-protein coupled receptor (GPCR) activity are the two significantly enriched GO functional categories in FS cases. Further analysis revealed a big overlap of genes between these two categories, 81.4% of the analyzed genes involved in GPCR activity also have the same GO terms in olfactory receptor activity. In other words, the majority of the GPCR-related genes make up the olfactory receptor activity. The genetic variation in human olfactory receptors, one of the largest gene families in humans, has been linked to phenotypic diversity^42^. The sense of smell is a complex process and requires a large number of olfactory receptors to differentiate minute differences among thousands of combinations of chemicals with differing concentrations^43^. Enrichment of olfactory-related genes for FS indels have been reported previously from investigation of genetic variation in an individual human exome and a systematic survey of loss of function (LoF) variants in human protein-coding genes^6, 28, 44^. Another study comparing human and chimpanzee olfactory receptor gene repertoires suggested that these genes are under relaxed selection, which may explain the relatively large number of variants in olfactory genes^45^.

**Figure 6:**
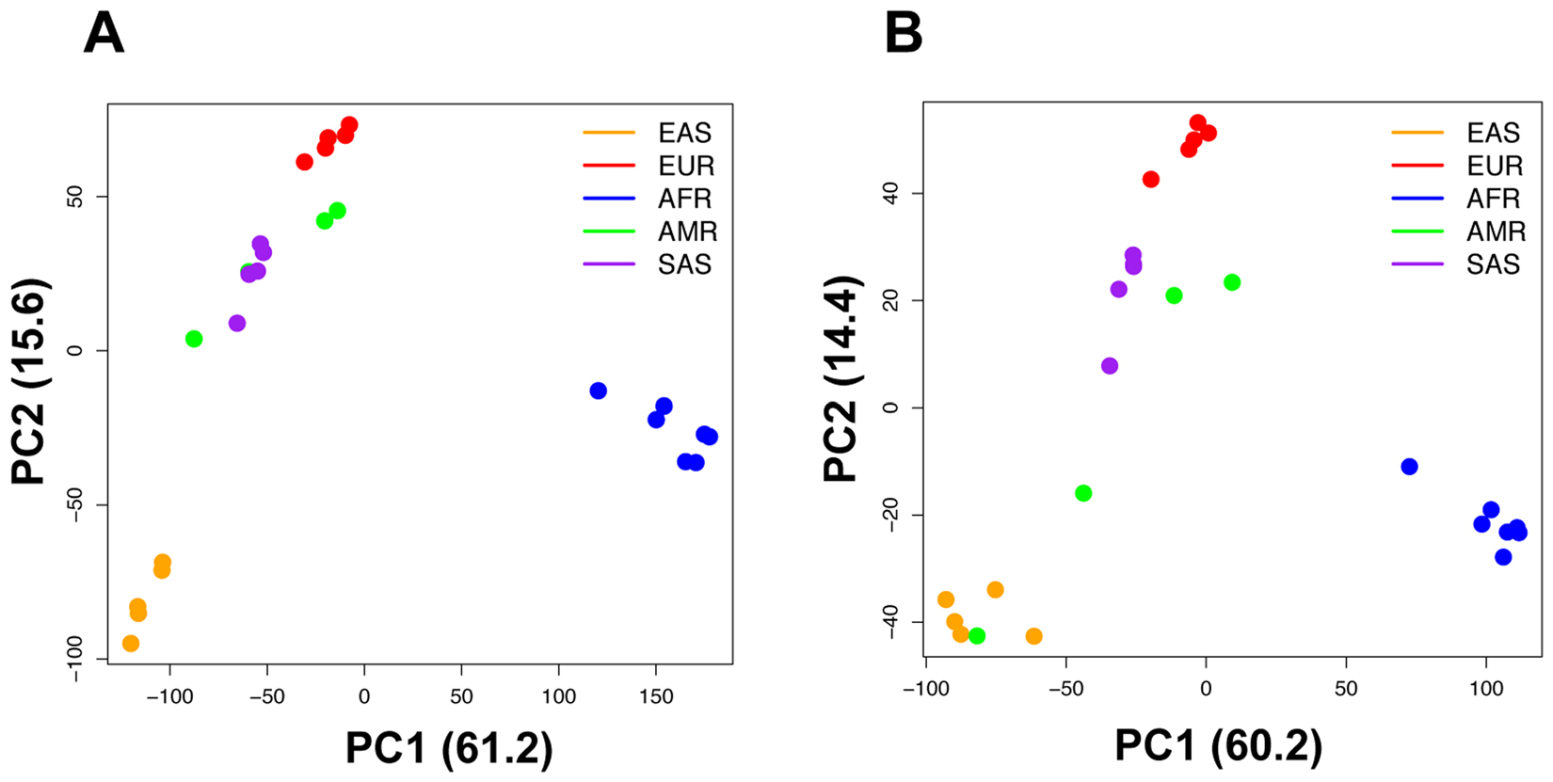
PCA analysis of transcription-related indel patterns in 26 populations. (**A**) All indels; (**B**) Homozygous indels only

The NFS enrichment analysis indicated an overrepresentation of transcription-related coding indels from 2,504 individual genomes. This is consistent with previous studies that demonstrated high variations in transcription-related genes and their potential link to phenotypic diversity^46, 47^. Ribeiro-dos-Santos *et al*. characterized transcription-related genes that have been the target of positive evolutionary forces^46^. In addition to describing a similar enrichment of transcription-related indels and their possible role in positive selection, Chen *et al*. suggested that these indels may contribute to the diversity of RNA and protein levels in humans, which gives rise to our unique traits^47^. The effects of these transcription-related NFS indels on protein structure and function are discussed in the next subsection. We also performed PCA analysis using genes with transcription-related indels only. There are 405 unique genes with 496 unique indels (322 deletions and 174 insertions) in transcription-related coding regions. Similar to the full indel data analysis, PCA analysis showed similar distinct clustering into the five super populations (Fig. 6). The AMR populations are less separated from SAS and EAS in the homozygous transcription-related indel analysis (Fig. 6B) than the all transcription-related indel analysis (Fig. 6A). This may be caused by a combination of two factors: a low number of homozygous transcription-related indels and the admixture of populations as discussed in investigation of human structural variants^23^.

Since small coding indels in the 1000 Genome Project have different allele frequency, one interesting question is if there are any differences in functional enrichment between common (≥ 1% allele frequency, about 32–37% of all indels) and rare indels (<1% allele frequency). Functional enrichment analysis showed similar results between the common and rare indels, *i.e*. NFS indels are significantly enriched (*p-value* < 0.05) in transcription-related genes and FS indels are enriched in olfactory-related genes and activities (Supplementary Figure S1).

### Effects of short NFS indels on protein structure

**Figure 7:**
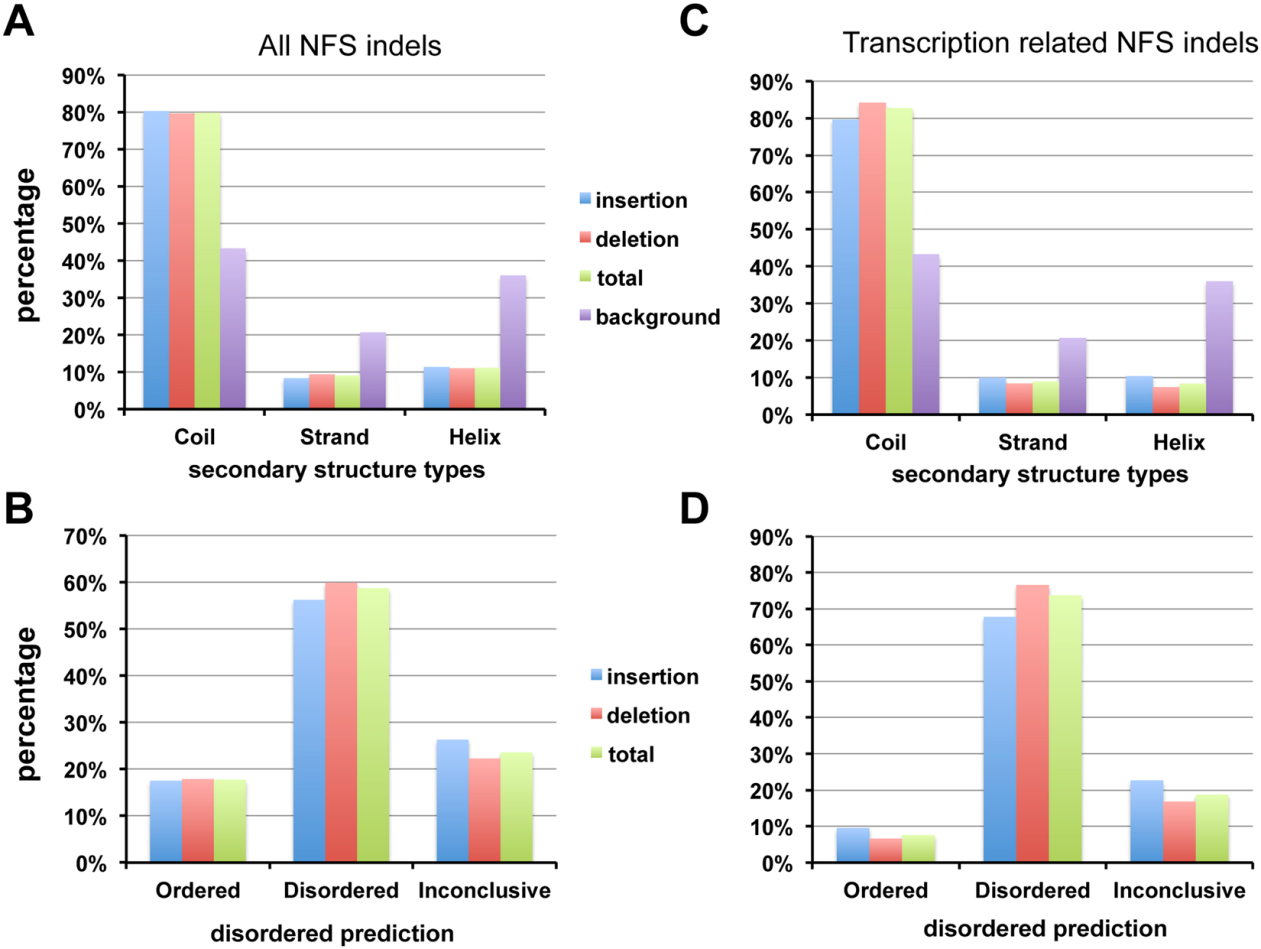
Secondary structure and residue disorder types for NFS indels. (**A**) Distribution of secondary structure types of all NFS indels; (**B**) Distribution of secondary structure types of transcription-related NFS indels; (**C**) Distribution of residue disorder of all NFS indels; (**D**) Distribution of residue disorder of transcription-related NFS indels. A residue in an indel is considered “disordered” or “ordered” if both IUPred and DisProt agree; otherwise it is annotated as “inconclusive”.

Due to the low number of matches of coding indels to known protein structures in PDB (79 NFS deletions and 12 NFS insertions)^33^, the secondary structure types of the remaining NFS indels were predicted as described in Methods. These coding NFS indels are depleted in the two regular major secondary structure types, helix (11%) and strand (9%), and highly enriched in coil conformation (80%) when compared to the background secondary structure type distribution as we reported previously (helix: 36%, strand: 20.8%, and coil: 43.2%, *p*-value of chi-square test <2.2 × 10^−16^)^35^ (Fig. 7A). There is no clear difference between NFS deletions and NFS insertions regarding their effect on secondary structures of proteins. Disordered residue prediction showed a similar pattern to that of secondary structure types for these NFS indels. Only about 20% of the NFS indel residues are predicted as ordered while about 60% of the residues are predicted as disordered (Fig. 7B). These results are consistent with our previous structural analysis of “natural” indels in PDB and the published work by Zhao *et al*., which showed a depleted regular secondary structure types (helix and strand) and highly enriched in disorder and coil conformation^31, 35^.

Since transcription-related genes are enriched in NFS indels, we examined the secondary structure types and disorder prediction of these indels to see if there are any significant differences between transcription-related and all NFS indels. In terms of secondary structure types, there are more coil types and fewer helix and strand conformations (*p*-value of chi-square test is 0.002) (Fig. 7A,C). Moreover, the disorder prediction is significantly different in transcription-related NFS indels compared to all NFS indels (*p*-value of chi-square test <2.2 × 10^−16^). There are more disordered residues in transcription-related NFS indels (Fig. 7B,D). The above results suggest that these transcription-related indels may keep the core of transcription-related proteins intact while introducing variations at the coil regions, providing differences in DNA binding affinity/specificity and contributes to phenotypic diversity^48, 49^. Binding differences have been shown to correlate well with differences in gene expression, which is a driving force in the evolution of organisms and plays an important role in phenotypic diversity^50–52^.

**Figure 8:**
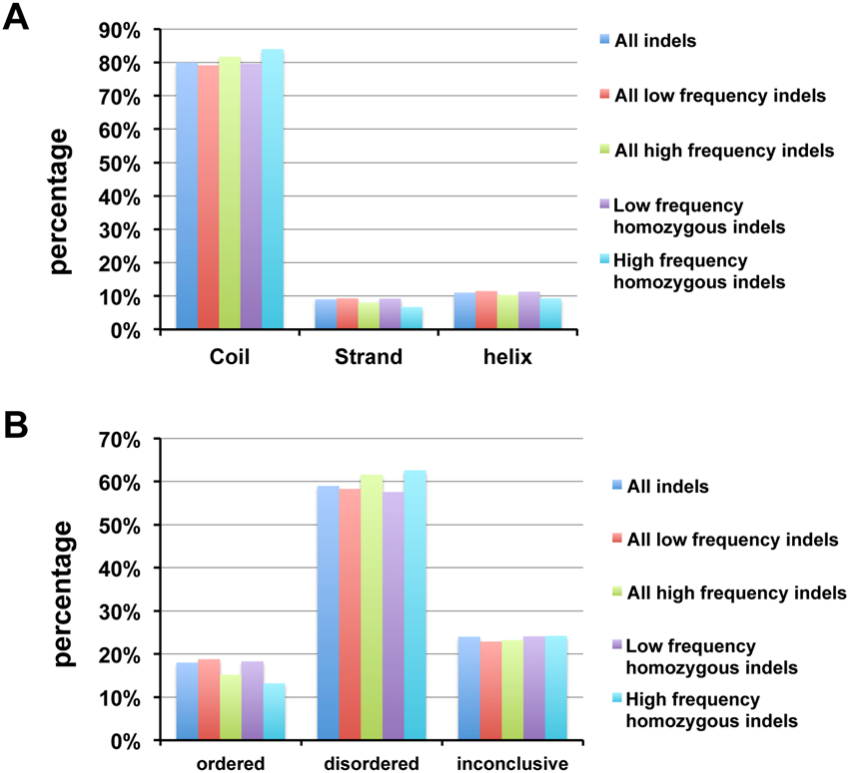
Comparisons of structural types between high and low allele frequency NFS indels. (**A**) Distribution of secondary structure types; (**B**) Distribution of residue disorder. A residue in an indel is considered “disordered” or “ordered” if both IUPred and DisProt predictions agree. Otherwise it is annotated as “Inconclusive”.

The structural effects were also compared between common and rare NFS indels. High frequency common NFS indels tend to have slightly more coil/disordered residues and fewer ordered residues including helix and strand secondary structure types than the rare NFS indels (Fig. 8). There are bigger differences between insertion and deletion indel types in common indels than those in rare indels, especially in homozygous NFS indels (Supplementary Figures S2 and S3). However, caution should be taken about these small differences, as they could be well within the prediction errors in protein secondary structure and disorder predictions.

Taken together, NFS indels from the general population genomes tend to locate on non-core structural segments and may have minimal effect on protein structural integrity. The deletion and insertion of small fragments in the coil region may result in differences in binding affinity and gene expression, which in turn can drive evolution and contribute to the diversity of phenotypes^48, 49^.

## Conclusion

Accurate prediction of structural and functional effects of indels, the second largest type of genetic variation in human genomes, is of paramount importance in interpretation of variation in genomes from various diseases. In this study, we analyzed all short indels in coding regions on chromosome 1–22 and chromosome X from the 1000 Genomes Projects to establish baseline characteristics of short coding indels in general, non-diseased populations. We found that these short NFS and FS indels are more likely to occur in N- and C-terminal regions and assume coil or disordered conformations. For the functional effects, FS indels are highly enriched in olfactory receptors while NFS indels are mainly associated with transcription-related functionalities.

FS coding indels are considered more deleterious as they change protein sequences and may result in loss-of-function variants for essential proteins. It is not surprising that the number of short FS coding indels is smaller than expected, as deleterious mutants are more likely to be removed from the population by natural selection. FS indels found in healthy individuals generally are less deleterious and contribute to phenotypic diversity through different ways. First, a second FS indel may rescue potential deleterious effect of the first FS indel by correcting the open reading frame (see Results section). Secondly, the protein with an FS indel might be non-essential or has other similar proteins to carry out the same function. Thirdly, a heterozygous FS indel has a normal copy of the gene to carry out the essential function unless the variant is dominant. Lastly, mis-annotations on the human reference genome also contribute to some of the FS indel cases, especially for the 100% frequency of homozygous FS indels (Supplementary Table S3).

## Methods

### Dataset

Raw variant call format (.vcf) files of the 1000 Genomes Project phase 3, including variant calls of chromosome 1 to 22 and chromosome X, were downloaded from the 1000 Genomes Project at http://www.1000genomes.org. All variants were annotated based on the coordinates of these variants with Variant Effect Predictor^53^. Since the goal of this study was to study the effect of indels on protein structure and function, we only selected insertions and deletions in coding regions, including frameshift and non-frameshift indels.

### Indel distribution and gene enrichment analysis

Indels’ distribution in 26 populations was analyzed. In counting the number of unique indels, the same indel occurring in multiple genomes was counted as 1. The relative frequency of each indel of a gene in each population was calculated, and the indel population patterns were visualized using PCA to identify if geographical and ancestral backgrounds can account for the distribution of coding indels. We also performed PCA on the homozygous only indels.

To investigate the functional categories of genes affected by these small indels, we applied DAVID 6.8, (the Database for Annotation, Visualization and Integrated Discovery) to perform functional enrichment analysis^40^. Lists of FS genes and NFS genes were analyzed separately. A cutoff of 0.05 was set for FDR (False discovery rate) to identify the significantly enriched functional categories.

### Protein structural analysis

To avoid redundancy, only protein sequence derived from the longest transcript was selected, which was downloaded from Ensembl’s FTP site (http://grch37.ensembl.org/info/data/ftp/index.html). Since FS indels change the amino acid sequences starting at the indel sites, we only performed structural analysis on NFS indels. The protein sequences with indels were first blasted against protein sequences *pdbaanr* with known structures in Protein Data Bank (PDB)^33, 54^. The alignments that had E-values less than 0.001 with at least 80% sequence identity and 50% coverage were selected. The secondary structure types of the deletion sequences with a reference protein structure were assigned using DSSP^55^. For protein sequences with indels that did not have corresponding protein structures available and insertion sequences that did not have corresponding secondary structures, the secondary structure types were predicted with RaptorX-SS8, an 8-class secondary structure prediction method^56^. Each indel residue was assigned to one of four secondary structure states, helix, strand, coil and disordered. DSSP program was used to assign three secondary structure states: helix, strand and coil following the widely used convention, H (α-helix), G (3_10_-helix) and I (π-helix) from DSSP as helix type; E (extended strand) and B (residue in isolated β-bridge) states as strand type and all the other states from DSSP are considered as coil^35^. The disordered residues were defined by comparing the “ATOM” and “SEQRES” records in PDB file. If a residue or a fragment appeared in “SEQRES”, but is missing from the “ATOM” record in a PDB file, this residue or fragment was considered disordered or unstructured^57^. Disorder predictions of indel residues were performed using IUPred^58^ and DisProt^59^.

### Data availability

The data used in this study were downloaded from the 1000 Genomes Project.

## Electronic supplementary material

Supplementary Data
