# Supplementary material for: Effects of short indels on protein structure and function in human genomes
Source: Sci Rep. 2017 Aug 24;7:9313. doi: 10.1038/s41598-017-09287-x (PMC5570956; doi:10.1038/s41598-017-09287-x)
Supplement: Supplementary file 1 — Supplementary Data [file 41598_2017_9287_MOESM1_ESM.pdf]

Supplementary data

Effects of short indels on protein structure and function in human genomes

Maoxuan Lin, Sarah Whitmire, Jing Chen, Alvin Farrel, Xinghua Shi, Jun-tao Guo*

Department of Bioinformatics and Genomics, The University of North Carolina at Charlotte, Charlotte, NC 28223, USA

Figure S1. Functional enrichment analysis of genes having NFS (A and B) or FS (C and D) indels with either high allele frequency (≥ 1%) or low allele frequency (< 1%). Some genes have indels of both high and low allele frequencies (258 and 207 genes for NFS and FS indels respectively). Significantly enriched (p-values < 0.05) categories are shown in terms of Biological Process (A and C) and Molecular Function (B and D).

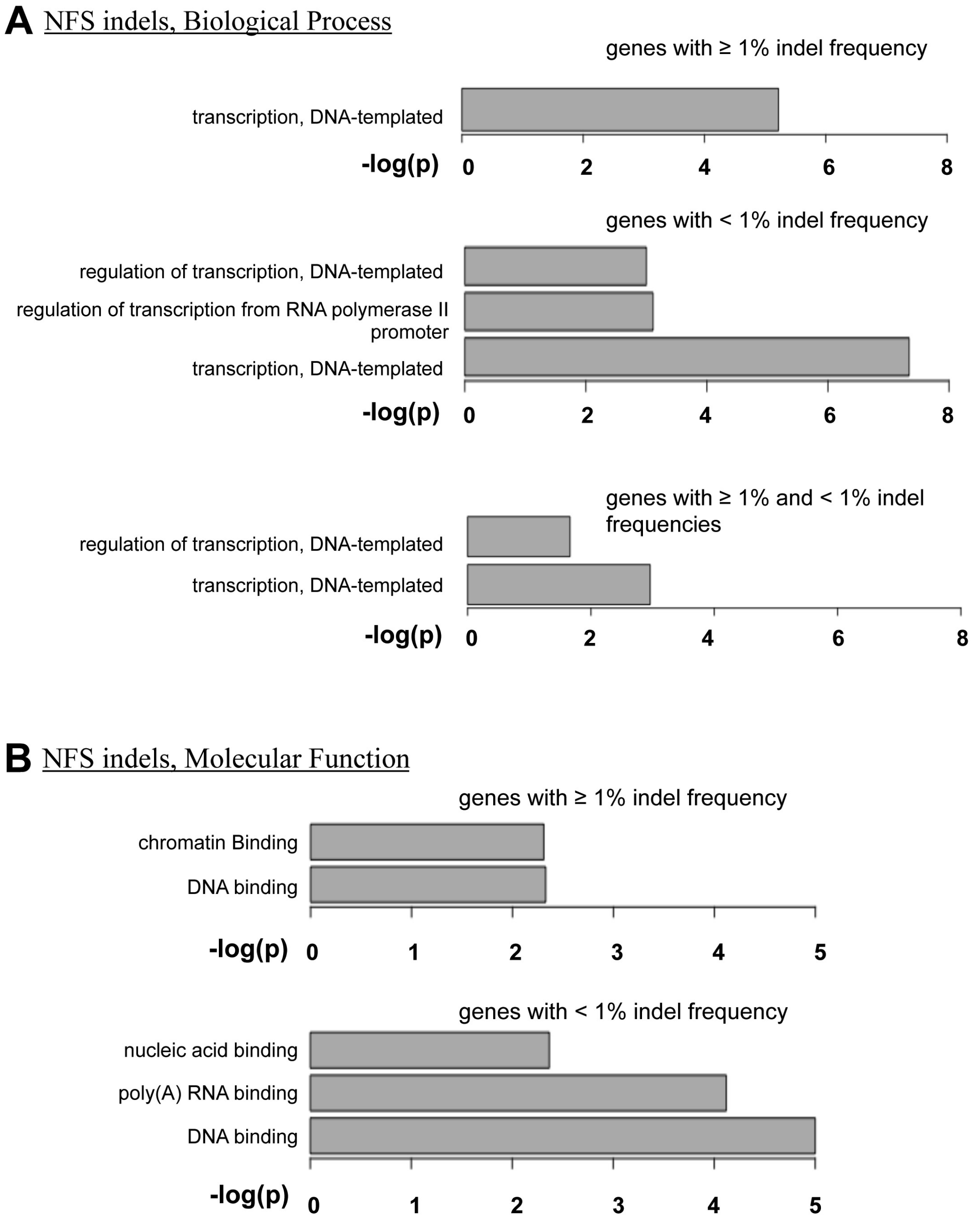

C FS indels, Biological Process

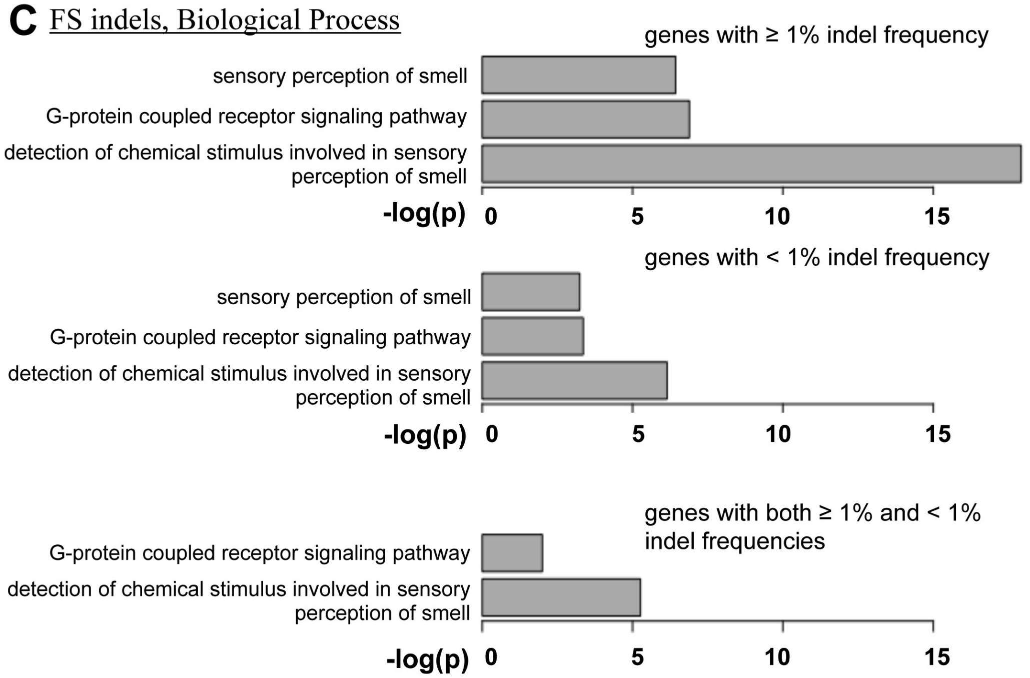

D FS indels, Molecular Function

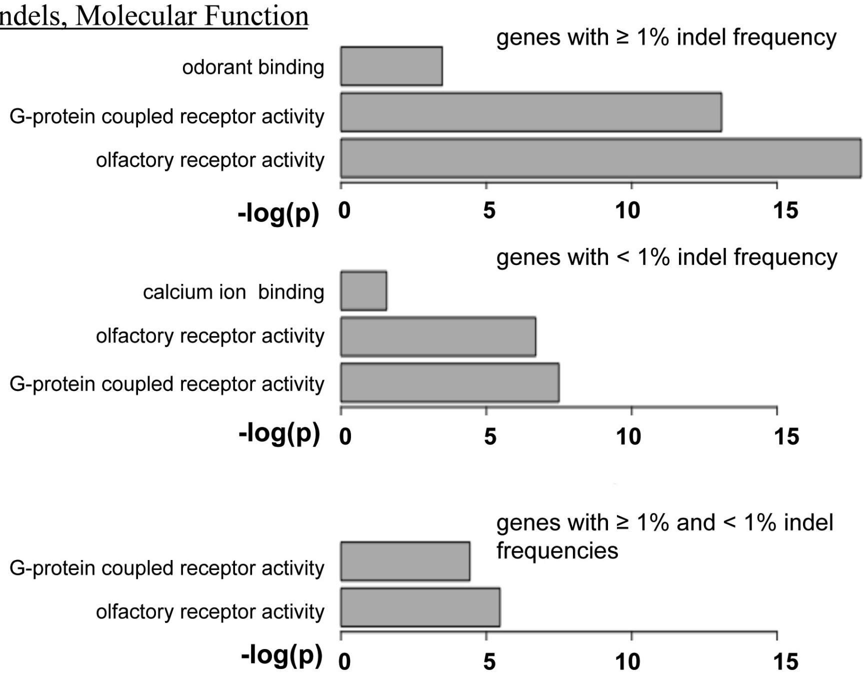

Figure S2. Comparisons of secondary structure types between high and low allele frequency NFS indels and indel types (insertion or deletion). (A) Distribution of all low frequency NFS indels; (B) Distribution of all high frequency NFS indels; (C) Distribution of homozygous low frequency NFS indels; (B) Distribution of homozygous high frequency NFS indels;

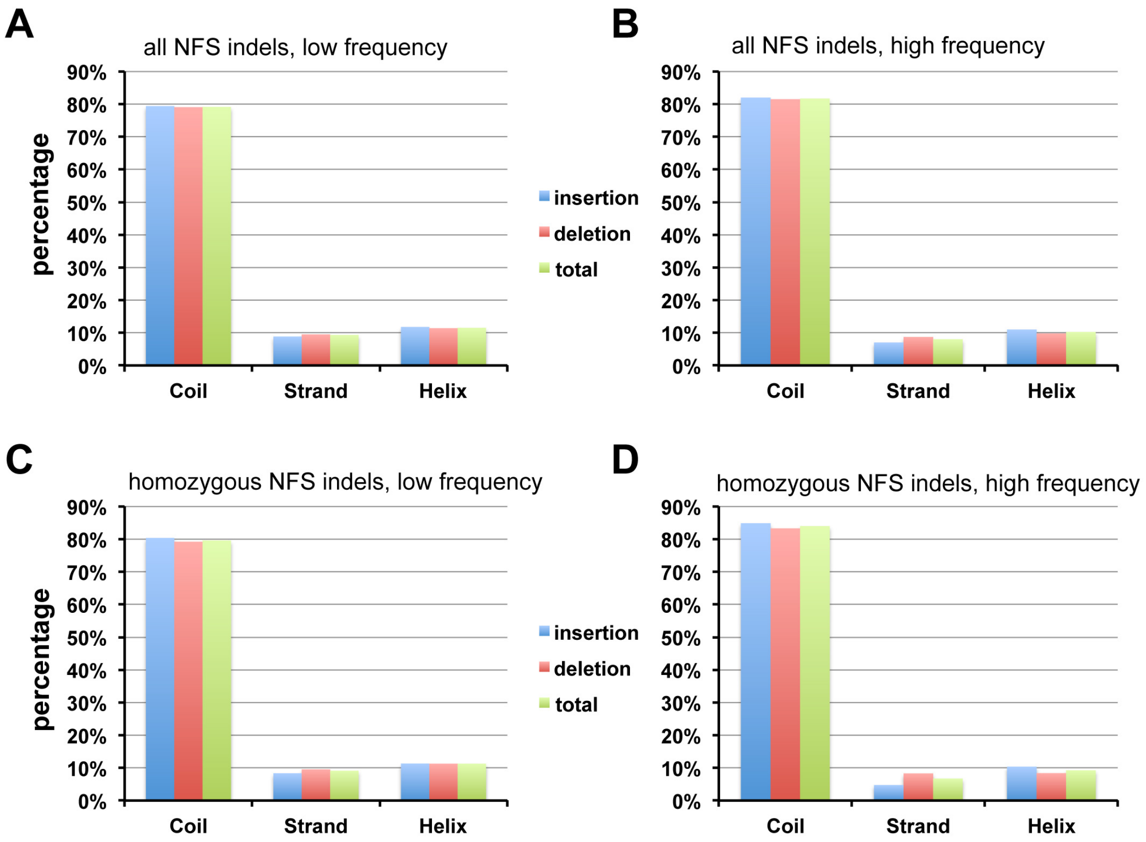

Figure S3. Comparisons of residue disorder prediction between high and low allele frequency NSF indels and indel types (insertion or deletion). (A) Distribution of all low frequency NFS indels; (B) Distribution of all high frequency NFS indels; (C) Distribution of homozygous low frequency NFS indels; (B) Distribution of homozygous high frequency NFS indels. A residue in an indel is considered “disordered” or “ordered” if both IUPred and DisProt agree; otherwise it is annotated as “inconclusive”.

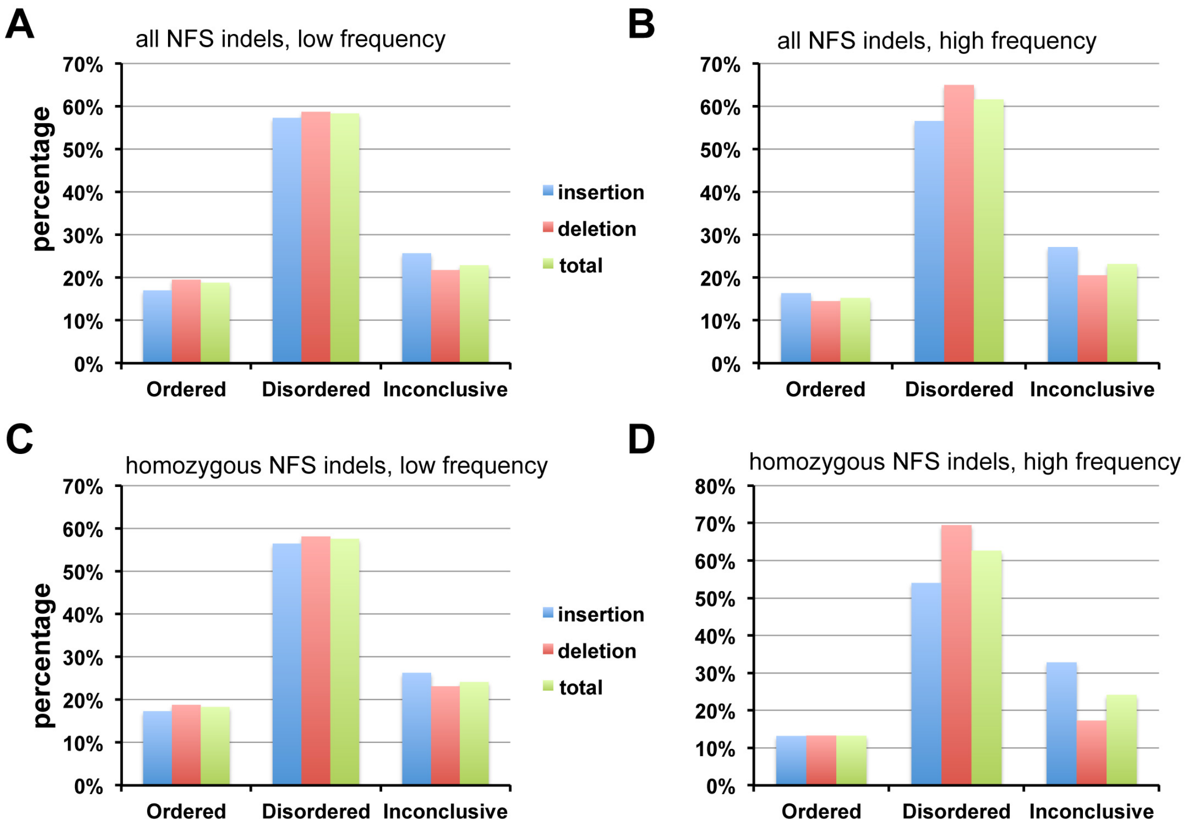

Table S1. High frequency homozygous indels among 2,504 individuals

Frequency	Number of NFS indels (#genes)	Number of FS indels (#genes)
>50%	72(72)	137(118)
>60%	58(58)	122(103)
>70%	44(44)	110(93)
>80%	33(33)	106(90)
>90%	28(28)	101(85)
100%	1(1)	61(54)

Table S2. Genes with NFS indels in more than 90 percent of the populations in the1000 Genome Project. A deletion in *TAF4* (underlined) appears in all genomes.

Ensembl_ID	Gene	Strand	Chr.	Position	Type	Length(bp)	Description
ENSG00000141449	GREB1L	1	18	19100758	Insertion	3	growth regulation by estrogen in breast cancer-like
ENSG00000130699	TAF4	-1	20	60640305	Deletion	6	TAF4 RNA polymerase II, TBP-associated factor
ENSG00000176731	C8orf59	-1	8	86126827	Insertion	6	chromosome 8 open reading frame 59
ENSG00000196782	MAML3	-1	4	140651584	Deletion	3	mastermind-like 3 (Drosophila)
ENSG00000185442	FAM174B	-1	15	93198678	Deletion	6	family with sequence similarity 174, member B
ENSG00000163067	ZNF2	1	2	95847040	Deletion	3	zinc finger protein 2
ENSG00000167311	ART5	-1	11	3661585	Insertion	3	ADP-ribosyltransferase 5
ENSG00000186564	FOXD2	1	1	47904668	Insertion	6	forkhead box D2
ENSG00000115457	IGFBP2	1	2	217498290	Insertion	9	insulin-like growth factor binding protein 2, 36kDa
ENSG00000160695	VPS11	1	11	118939939	Insertion	3	vacuolar protein sorting 11 homolog (S. cerevisiae)
ENSG00000157827	FMNL2	1	2	153476066	Insertion	3	formin-like 2
ENSG00000198598	MMP17	1	12	132313098	Insertion	9	matrix metallopeptidase 17
ENSG00000150630	VEGFC	-1	4	177605081	Deletion	3	vascular endothelial growth factor C
ENSG00000182500	ORAI1	1	12	122064773	Deletion	6	ORAI calcium release-activated calcium modulator 1
ENSG00000179532	DNHD1	1	11	6567895	Insertion	12	dynein heavy chain domain 1
ENSG00000162290	DCP1A	-1	3	53324819	Deletion	6	decapping mRNA 1A
ENSG00000204842	ATXN2	-1	12	112036753	Deletion	3	ataxin 2
ENSG00000150045	KLRF1	1	12	9994445	Deletion	3	killer cell lectin-like receptor subfamily F, member 1
ENSG00000186472	PCLO	-1	7	82581488	Insertion	3	piccolo presynaptic cytomatrix protein
ENSG00000176225	RTTN	-1	18	67863850	Insertion	3	rotatin
ENSG00000012223	LTF	-1	3	46501284	Insertion	3	lactotransferrin
ENSG00000003147	ICA1	-1	7	8196567	Deletion	18	islet cell autoantigen 1, 69kDa
ENSG00000267909	CCDC177	-1	14	70039803	Deletion	6	Homo sapiens coiled-coil domain containing 177 (CCDC177)
ENSG00000239704	CDRT4	-1	17	15343524	Insertion	3	CMT1A duplicated region transcript 4
ENSG00000196498	NCOR2	-1	12	124824721	Insertion	9	nuclear receptor corepressor 2
ENSG00000179588	ZFPM1	1	16	88599702	Deletion	3	zinc finger protein, FOG family member 1
ENSG00000173702	MUC13	-1	3	124646705	Insertion	3	mucin 13, cell surface associated
ENSG00000127529	OR7C2	1	19	15052983	Insertion	3	olfactory receptor, family 7, subfamily C, member 2

Table S3. Genes with FS indels appear in all the populations of the 1000 Genome Project

Ensembl_ID	Gene	Strand	Chr.	Position	Type	Length(bp)	Description
ENSG00000174450	GOLGA6L2	-1	15	23685541	Deletion	1	golgin A6 family-like 2
ENSG00000108819	PPP1R9B	-1	17	48227384	Insertion	2	protein phosphatase 1, regulatory subunit 9B
ENSG00000108819	PPP1R9B	-1	17	48227403	Insertion	2	protein phosphatase 1, regulatory subunit 9B
ENSG00000160957	RECQL4	-1	8	145738767	Deletion	1	RecQ protein-like 4
ENSG00000103148	NPRL3	-1	16	138772	Insertion	1	nitrogen permease regulator-like 3 (S. cerevisiae)
ENSG00000115204	MPV17	-1	2	27535311	Insertion	1	MpV17 mitochondrial inner membrane protein
ENSG00000147676	MAL2	1	8	120220775	Deletion	1	mal, T-cell differentiation protein 2 (gene/pseudogene)
ENSG00000159140	SON	1	21	34948684	Insertion	1	SON DNA binding protein
ENSG00000159140	SON	1	21	34948696	Deletion	1	SON DNA binding protein
ENSG00000182253	SYNM	1	15	99646107	Insertion	1	synemin, intermediate filament protein
ENSG00000085365	SCAMP1	1	5	77745853	Insertion	1	secretory carrier membrane protein 1
ENSG00000116031	CD207	-1	2	71062833	Insertion	1	CD207 molecule, langerin
ENSG00000178814	OPLAH	-1	8	145106938	Deletion	2	5-oxoprolinase (ATP-hydrolysing)
ENSG00000055163	CYFIP2	1	5	156721863	Insertion	1	cytoplasmic FMR1 interacting protein 2
ENSG00000169499	PLEKHA2	1	8	38827185	Deletion	1	pleckstrin homology domain containing, family A
ENSG00000004139	SARM1	1	17	26699195	Insertion	1	sterile alpha and TIR motif containing 1
ENSG00000004139	SARM1	1	17	26699367	Insertion	1	sterile alpha and TIR motif containing 1
ENSG00000185340	GAS2L1	1	22	29707639	Insertion	1	growth arrest-specific 2 like 1
ENSG00000137700	SLC37A4	-1	11	118898435	Deletion	1	solute carrier family 37 (glucose-6-phosphate transporter)
ENSG00000006534	ALDH3B1	1	11	67786064	Insertion	1	aldehyde dehydrogenase 3 family, member B1
ENSG00000076351	SLC46A1	-1	17	26727721	Deletion	1	solute carrier family 46 (folate transporter), member 1
ENSG00000181652	ATG9B	-1	7	150713902	Insertion	1	autophagy related 9B
ENSG00000090006	LTBP4	1	19	41123093	Insertion	1	latent transforming growth factor beta binding protein 4
ENSG00000154035	C17orf103	-1	17	21156530	Insertion	1	chromosome 17 open reading frame 103
ENSG00000258529	ALG9	-1	11	111742145	Deletion	1	Alpha-1,2-mannosyltransferase ALG9
ENSG00000150764	DIXDC1	1	11	111853106	Insertion	1	DIX domain containing 1
ENSG00000231274	SBK3	-1	19	56052337	Insertion	1	SH3 domain binding kinase family, member 3
ENSG00000113658	SMAD5	1	5	135513085	Insertion	1	SMAD family member 5
ENSG00000149294	NCAM1	1	11	112832339	Deletion	1	neural cell adhesion molecule 1
ENSG00000165862	PNLIPRP2	1	10	118383463	Insertion	1	pancreatic lipase-related protein 2
ENSG00000162040	HS3ST6	-1	16	1968338	Insertion	1	heparan sulfate (glucosamine) 3-O-sulfotransferase 6
ENSG00000167962	ZNF598	-1	16	2059621	Deletion	1	zinc finger protein 598
ENSG00000244486	SCARF2	-1	22	20779973	Insertion	1	scavenger receptor class F, member 2
ENSG00000244486	SCARF2	-1	22	20780024	Insertion	1	scavenger receptor class F, member 2
ENSG00000244486	SCARF2	-1	22	20780029	Insertion	1	scavenger receptor class F, member 2
ENSG00000129270	MMP28	-1	17	34100350	Insertion	1	matrix metallopeptidase 28
ENSG00000166333	ILK	1	11	6630028	Deletion	1	integrin-linked kinase
ENSG00000173566	NUDT18	-1	8	21966700	Deletion	1	nudix (nucleoside diphosphate linked moiety X)-type motif 18
ENSG00000173566	NUDT18	-1	8	21966707	Deletion	1	nudix (nucleoside diphosphate linked moiety X)-type motif 18
ENSG00000110057	UNC93B1	-1	11	67765163	Insertion	1	unc-93 homolog B1 (C. elegans)
ENSG00000106631	MYL7	-1	7	44180079	Insertion	1	myosin, light chain 7, regulatory
ENSG00000171606	ZNF274	1	19	58718360	Insertion	1	zinc finger protein 274
ENSG00000004142	POLDIP2	-1	17	26684390	Insertion	1	polymerase (DNA-directed), delta interacting protein 2
ENSG00000042317	SPATA7	1	14	88852279	Deletion	1	spermatogenesis associated 7
ENSG00000253729	PRKDC	-1	8	48805816	Insertion	1	protein kinase, DNA-activated, catalytic polypeptide
ENSG00000101493	ZNF516	-1	18	74090957	Deletion	1	zinc finger protein 516
ENSG00000139737	SLAIN1	1	13	78272276	Insertion	1	SLAIN motif family, member 1
ENSG00000167984	NLRC3	-1	16	3602227	Deletion	1	NLR family, CARD domain containing 3
ENSG00000211852	TRAJ37	1	14	22972783	Deletion	1	T cell receptor alpha joining 37
ENSG00000211853	TRAJ36	1	14	22974150	Deletion	1	T cell receptor alpha joining 36
ENSG00000019144	PHLDB1	1	11	118505676	Deletion	1	pleckstrin homology-like domain, family B, member 1
ENSG00000152910	CNTNAP4	1	16	76311602	Insertion	1	contactin associated protein-like 4
ENSG00000272333	KMT2B	1	19	36214632	Insertion	1	Histone-lysine N-methyltransferase 2B
ENSG00000167595	C19orf55	1	19	36258937	Deletion	1	chromosome 19 open reading frame 55
ENSG00000186806	VSIG10L	-1	19	51835892	Insertion	1	V-set and immunoglobulin domain containing 10 like
ENSG00000160963	COL26A1	1	7	101200669	Insertion	1	collagen, type XXVI, alpha 1
ENSG00000198488	B3GNT6	1	11	76751541	Deletion	1	betaGal beta-1,3-N-acetylglucosaminyltransferase 6
ENSG00000198488	B3GNT6	1	11	76751584	Deletion	1	betaGal beta-1,3-N-acetylglucosaminyltransferase 6
ENSG00000198488	B3GNT6	1	11	76751603	Deletion	1	betaGal beta-1,3-N-acetylglucosaminyltransferase 6
ENSG00000196684	HSH2D	1	19	16268207	Deletion	1	hematopoietic SH2 domain containing
ENSG00000008735	MAPK8IP2	1	22	51042513	Insertion	1	mitogen-activated protein kinase 8 interacting protein 2
ENSG00000110811	LEPREL2	1	12	6938023	Insertion	1	leprecan-like 2

Table S4. List of genes with at least two FS indels appear on the same chromosome from the same individual genome.

Gene	Strand	Ensembl_ID	Chromosome	# of individuals	Position	Indel Type	Length(bp)
SARM1	1	ENSG00000004139	17	2504	26699195	Insertion	1
					26699199	Insertion	1
					26699367	Insertion	1
COPZ2	-1	ENSG00000005243	17	2504	46115072	Insertion	1
					46115122	Insertion	1
					46115084	Insertion	2
ALDH3B1	1	ENSG00000006534	11	14	67786064	Insertion	1
					67789291	Insertion	1
CYP3A43	1	ENSG00000021461	7	50	99434077	Deletion	1
					99463552	Deletion	1
IKZF2	-1	ENSG00000030419	2	31	214012404	Insertion	1
					214012405	Insertion	1
SERPINB3	-1	ENSG00000057149	18	257	61323012	Insertion	1
					61323011	Insertion	2
CTSA	1	ENSG00000064601	20	202	44520257	Deletion	1
					44520260	Deletion	1
					44520257	Deletion	2
					44520259	Deletion	2
SPI1	-1	ENSG00000066336	11	3	47380218	Deletion	4
					47380276	Insertion	2
PHRF1	1	ENSG00000070047	11	16	608506	Deletion	10
					608517	Deletion	2
CLTCL1	-1	ENSG00000070371	22	27	19170999	Deletion	10
					19171074	Insertion	1
					19189003	Insertion	1
CPSF1	-1	ENSG00000071894	8	11	145623003	Deletion	2
					145622990	Deletion	7
ATP12A	1	ENSG00000075673	13	16	25266675	Deletion	1
					25266666	Deletion	4
EPDR1	1	ENSG00000086289	7	2	37960262	Deletion	13
					37960406	Deletion	5
VRK1	1	ENSG00000100749	14	35	97327135	Deletion	2
					97327098	Insertion	2
MROH8	-1	ENSG00000101353	20	28	35740759	Deletion	1
					35807797	Deletion	2
					35807790	Insertion	29
					35807790	Insertion	29
CEP192	1	ENSG00000101639	18	64	13049811	Deletion	1
					13049813	Deletion	2
SLC25A14	1	ENSG00000102078	X	3	129493076	Insertion	4
					129493077	Insertion	4
DHDH	1	ENSG00000104808	19	246	49447748	Deletion	2
					49442849	Insertion	1
HBP1	1	ENSG00000105856	7	36	106826381	Insertion	1
					106826380	Insertion	2
PITRM1	-1	ENSG00000107959	10	2	3181184	Deletion	2
					3208567	Insertion	25
CYP27B1	-1	ENSG00000111012	12	6	58162765	Deletion	2
					58162768	Insertion	1
MYF5	1	ENSG00000111049	12	4	81112731	Deletion	1
					81112733	Deletion	2
DUSP16	-1	ENSG00000111266	12	456	12630681	Deletion	4
					12630675	Deletion	5
TIMELESS	-1	ENSG00000111602	12	9	56817449	Insertion	1
					56817451	Insertion	2
HSPA9	-1	ENSG00000113013	5	1	137896580	Insertion	1
					137896597	Insertion	1
ACADL	-1	ENSG00000115361	2	9	211068109	Deletion	2
					211068107	Insertion	1
					211068105	Insertion	8
CD207	-1	ENSG00000116031	2	52	71062870	Deletion	1
					71060821	Deletion	2
					71062833	Insertion	1
GRIN3B	1	ENSG00000116032	19	4	1004589	Deletion	2
					1007860	Insertion	1
					1004896	Insertion	4
NRP2	1	ENSG00000118257	2	123	206641239	Deletion	4
					206641245	Insertion	1

OBP2A	1	ENSG00000122136	9	7	138439733	Deletion	2
					138441685	Insertion	1
TNFAIP6	1	ENSG00000123610	2	3	152236045	Deletion	1
					152235992	Insertion	1
SOX4	1	ENSG00000124766	6	9	21595266	Insertion	1
					21595263	Insertion	2
AMOT	-1	ENSG00000126016	X	2	112022627	Deletion	1
					112022655	Deletion	1
					112022650	Deletion	4
PIN1	1	ENSG00000127445	19	5	9948698	Deletion	1
					9948693	Deletion	2
PODXL	-1	ENSG00000128567	7	25	131241045	Deletion	1
					131241039	Deletion	5
TTI2	-1	ENSG00000129696	8	5	33370003	Deletion	1
					33370000	Deletion	2
STK33	-1	ENSG00000130413	11	46	8414089	Insertion	1
					8414090	Insertion	5
HRC	-1	ENSG00000130528	19	15	49657740	Deletion	1
					49657742	Deletion	2
OLFM1	1	ENSG00000130558	9	392	137968894	Deletion	1
					137968914	Insertion	2
					137968919	Insertion	2
					137968996	Insertion	2
PNPLA7	-1	ENSG00000130653	9	1	140373565	Deletion	1
					140417185	Deletion	5
					140356449	Insertion	1
					140437144	Insertion	1
					140444627	Insertion	1
EPSTI1	-1	ENSG00000133106	13	12	43463359	Insertion	1
					43462434	Insertion	5
AGAP3	1	ENSG00000133612	7	2504	150783900	Insertion	1
					150783908	Insertion	1
CHL1	1	ENSG00000134121	3	185	239347	Insertion	1
					239555	Insertion	1
DSG3	1	ENSG00000134757	18	10	29054284	Deletion	1
					29054289	Deletion	2
TROAP	1	ENSG00000135451	12	19	49724075	Deletion	1
					49724077	Deletion	8
AOAH	-1	ENSG00000136250	7	2504	36552729	Insertion	1
					36552790	Insertion	1
IGFBPL1	-1	ENSG00000137142	9	48	38411403	Deletion	7
					38411415	Insertion	1
C4orf17	1	ENSG00000138813	4	99	100443781	Deletion	1
					100451038	Deletion	10
					100451040	Deletion	11
					100451049	Deletion	2
					100460420	Insertion	1
CELA1	-1	ENSG00000139610	12	1352	51735071	Deletion	1
					51740414	Deletion	2
					51723598	Insertion	1
					51740413	Insertion	1
CTRL	-1	ENSG00000141086	16	5	67964623	Deletion	2
					67964622	Insertion	1
OR4D1	1	ENSG00000141194	17	69	56232679	Deletion	2
					56233444	Insertion	1
ANP32E	-1	ENSG00000143401	1	111	150195603	Deletion	1
					150195609	Insertion	1
FLG	-1	ENSG00000143631	1	1	152279416	Deletion	1
					152283937	Deletion	1
					152284040	Deletion	1
					152285076	Deletion	4
					152280404	Deletion	8
					152284886	Insertion	17
VWDE	-1	ENSG00000146530	7	76	12381721	Deletion	1
					12391268	Insertion	1
					12417523	Insertion	1
ZAN	1	ENSG00000146839	7	14	100364851	Deletion	1
					100391838	Deletion	1
					100346026	Deletion	2
					100385561	Deletion	35
					100371476	Insertion	1
					100377138	Insertion	4
NCAM1	1	ENSG00000149294	11	2504	112832339	Deletion	1

					112832276	Insertion	1
					112832307	Insertion	1
PLOD2	-1	ENSG00000152952	3	36	145803137	Insertion	1
					145803138	Insertion	1
ABCA10	-1	ENSG00000154263	17	816	67145190	Deletion	2
					67184000	Deletion	2
					67190117	Deletion	2
					67181671	Deletion	20
					67190536	Deletion	4
					67161166	Deletion	5
					67150465	Insertion	8
CNTNAP3B	1	ENSG00000154529	9	10	43816775	Deletion	1
					43844264	Deletion	1
					43844307	Deletion	4
MPPE1	-1	ENSG00000154889	18	4	11886988	Deletion	1
					11886987	Insertion	2
LAD1	-1	ENSG00000159166	1	383	201356001	Deletion	1
					201356004	Deletion	2
ZNF66	1	ENSG00000160229	19	347	20973551	Insertion	1
					20973560	Insertion	1
MSLNL	-1	ENSG00000162006	16	369	825655	Deletion	1
					830559	Deletion	1
					830743	Deletion	1
					830745	Deletion	2
					820636	Insertion	1
					830766	Insertion	1
					830767	Insertion	2
HS3ST6	-1	ENSG00000162040	16	5	1967982	Insertion	1
					1968338	Insertion	1
RYK	-1	ENSG00000163785	3	2504	133969437	Insertion	1
					133969487	Insertion	1
TIGD6	-1	ENSG00000164296	5	50	149374879	Deletion	1
					149375720	Deletion	1
					149374775	Deletion	2
TMEM67	1	ENSG00000164953	8	103	94772148	Insertion	1
					94772206	Insertion	4
SKA3	-1	ENSG00000165480	13	1	21729838	Deletion	1
					21746600	Deletion	1
HTRA1	1	ENSG00000166033	10	27	124221202	Insertion	1
					124221201	Insertion	2
PKD1L2	-1	ENSG00000166473	16	551	81164183	Deletion	1
					81194421	Deletion	1
					81242148	Deletion	2
					81253916	Deletion	2
					81194328	Deletion	5
					81161473	Insertion	22
AC018755.1	-1	ENSG00000167765	19	52	52097396	Deletion	1
					52096033	Deletion	5
					52097560	Insertion	1
ZNF83	-1	ENSG00000167766	19	11	53116985	Deletion	1
					53116949	Insertion	1
					53116948	Insertion	2
ZNF598	-1	ENSG00000167962	16	2504	2059621	Deletion	1
					2059736	Deletion	1
DHRSX	-1	ENSG00000169084	X	1	2418591	Deletion	2
					2418620	Deletion	5
ZNF212	1	ENSG00000170260	7	19	148936895	Deletion	1
					148936917	Deletion	16
					148937011	Insertion	10
OR7G3	-1	ENSG00000170920	19	307	9236916	Deletion	1
					9236698	Insertion	5
FBXW10	1	ENSG00000171931	17	2	18681857	Deletion	1
					18647847	Deletion	2
					18653309	Insertion	1
					18653174	Insertion	4
OR8I2	1	ENSG00000172154	11	190	55861277	Deletion	1
					55861306	Deletion	1
MANEA	1	ENSG00000172469	6	130	96034895	Deletion	2
					96034881	Insertion	2
					96034881	Insertion	4
					96034881	Insertion	4
					96034869	Insertion	5
					96034881	Insertion	8

					96034881	Insertion	8
C17orf66	-1	ENSG00000172653	17	3	34190017	Deletion	1
					34182099	Insertion	1
CD7	-1	ENSG00000173762	17	59	80274161	Deletion	1
					80274159	Insertion	1
OR4S2	1	ENSG00000174982	11	3	55418991	Deletion	1
					55418995	Deletion	1
TP53I11	-1	ENSG00000175274	11	4	44924950	Deletion	1
					44925373	Deletion	1
					44925359	Insertion	1
TSGA10IP	1	ENSG00000175513	11	15	65713196	Deletion	11
					65713170	Insertion	17
TPRN	-1	ENSG00000176058	9	5	140087040	Deletion	1
					140087043	Deletion	2
OR4L1	1	ENSG00000176246	14	68	20528600	Deletion	1
					20528448	Deletion	19
					20528397	Deletion	2
					20528880	Insertion	1
OR10AC1P	-1	ENSG00000176510	7	9	143208084	Deletion	1
					143208737	Deletion	1
MUC20	1	ENSG00000176945	3	6	195452663	Deletion	1
					195453017	Deletion	1
					195456537	Deletion	1
					195453042	Deletion	2
OR2T12	-1	ENSG00000177201	1	1	248458875	Deletion	1
					248458132	Deletion	2
					248458629	Deletion	5
OR2AJ1	1	ENSG00000177275	1	8	248097335	Deletion	1
					248097842	Deletion	1
					248097443	Deletion	4
					248098037	Deletion	4
ACOT4	1	ENSG00000177465	14	2	74060517	Insertion	1
					74060511	Insertion	4
IRX3	-1	ENSG00000177508	16	4	54317566	Insertion	1
					54317575	Insertion	1
					54317583	Insertion	1
TMEM78	1	ENSG00000177800	1	1	229385667	Deletion	2
					229385669	Deletion	2
					229385594	Deletion	4
					229385720	Deletion	4
BET1L	-1	ENSG00000177951	11	16	180317	Insertion	1
					180318	Insertion	1
PDE4DIP	-1	ENSG00000178104	1	744	144873962	Deletion	1
					144917827	Deletion	1
					144923728	Deletion	1
RNF212	-1	ENSG00000178222	4	2324	1087327	Insertion	17
					1087328	Insertion	2
					1087329	Insertion	2
FAM132B	1	ENSG00000178752	2	2	239072419	Deletion	1
					239067797	Insertion	8
ARID3B	1	ENSG00000179361	15	8	74836316	Insertion	1
					74836315	Insertion	2
ZFPM1	1	ENSG00000179588	16	2495	88599700	Deletion	1
					88599695	Deletion	2
					88600697	Insertion	1
CRIPAK	1	ENSG00000179979	4	582	1388672	Deletion	1
					1388922	Deletion	1
					1388344	Deletion	2
					1388409	Deletion	2
					1388503	Deletion	2
					1388562	Deletion	2
					1388713	Deletion	2
					1388744	Deletion	2
					1388899	Deletion	2
					1388961	Deletion	2
					1389146	Deletion	2
					1389360	Deletion	2
					1389424	Deletion	2
					1388569	Deletion	5
					1388375	Insertion	2
					1388530	Insertion	2
					1388622	Insertion	2
					1388682	Insertion	2

					1388930	Insertion	2
					1389117	Insertion	2
PSMG4	1	ENSG00000180822	6	4	3255401	Deletion	1
					3255405	Deletion	1
OR52D1	1	ENSG00000181609	11	9	5510498	Insertion	1
					5510540	Insertion	4
PTCHD3	-1	ENSG00000182077	10	3	27687759	Deletion	1
					27702950	Deletion	1
					27687534	Insertion	1
					27702256	Insertion	1
					27702261	Insertion	1
					27703042	Insertion	1
TSPAN10	1	ENSG00000182612	17	3	79612504	Deletion	1
					79614932	Deletion	4
ZNF721	-1	ENSG00000182903	4	1	436992	Deletion	1
					435540	Deletion	2
					437947	Deletion	5
ABHD16B	1	ENSG00000183260	20	3	62494218	Deletion	2
					62494226	Insertion	1
					62493175	Insertion	23
C1QTNF8	-1	ENSG00000184471	16	7	1143523	Deletion	1
					1143508	Deletion	14
					1143978	Insertion	1
AHNAK2	-1	ENSG00000185567	14	4	105417462	Deletion	1
					105415409	Deletion	2
					105417468	Insertion	1
					105418452	Insertion	1
PDIA2	1	ENSG00000185615	16	1	336700	Deletion	2
					336462	Deletion	4
					336888	Insertion	1
					335584	Insertion	7
SPATA31A6	-1	ENSG00000185775	9	15	43627263	Insertion	5
					43627265	Insertion	7
PRSS38	1	ENSG00000185888	1	4	228033701	Insertion	2
					228033700	Insertion	4
WWOX	1	ENSG00000186153	16	2	79245892	Deletion	5
					79245873	Insertion	5
					79245998	Insertion	5
NUDT17	-1	ENSG00000186364	1	69	145586678	Deletion	1
					145586671	Deletion	5
C14orf23	1	ENSG00000186960	14	1167	29261309	Insertion	1
					29261306	Insertion	2
OR2T27	-1	ENSG00000187701	1	7	248813309	Insertion	1
					248813733	Insertion	1
PLA2G4E	-1	ENSG00000188089	15	17	42302330	Insertion	1
					42302338	Insertion	1
					42302330	Insertion	2
NUTM2G	1	ENSG00000188152	9	25	99694588	Deletion	1
					99694583	Deletion	2
ZNF626	-1	ENSG00000188171	19	7	20807935	Deletion	2
					20807177	Insertion	1
ANKDD1B	1	ENSG00000189045	5	130	74931681	Deletion	1
					74907504	Deletion	13
					74931676	Deletion	4
OR6K2	-1	ENSG00000196171	1	3	158669770	Deletion	2
					158670074	Deletion	2
					158669774	Insertion	1
OR4Q2	1	ENSG00000196383	14	9	20470852	Deletion	1
					20470582	Insertion	7
OR5AC2	1	ENSG00000196578	3	63	97806298	Deletion	1
					97806700	Insertion	1
MAML3	-1	ENSG00000196782	4	9	140811083	Deletion	10
					140811085	Deletion	11
					140811094	Deletion	2
OR2T4	1	ENSG00000196944	1	234	248525638	Deletion	1
					248524964	Deletion	2
					248525287	Deletion	4
					248525328	Insertion	2
ZNF860	1	ENSG00000197385	3	34	32030579	Deletion	1
					32031990	Deletion	2
					32031987	Insertion	1
					32031988	Insertion	1
HIST4H4	-1	ENSG00000197837	12	20	14923934	Insertion	1

					14923935	Insertion	1
PEG3	-1	ENSG00000198300	19	4	57335978	Deletion	1
					57335984	Deletion	1
					57328278	Deletion	2
					57335982	Insertion	2
RTP2	-1	ENSG00000198471	3	65	187416762	Deletion	1
					187416758	Insertion	1
OR2M2	1	ENSG00000198601	1	4	248343601	Deletion	1
					248343603	Deletion	2
MUC2	1	ENSG00000198788	11	67	1092801	Deletion	1
					1092503	Deletion	5
					1092669	Insertion	2
					1092799	Insertion	2
					1092804	Insertion	4
EFCAB2	1	ENSG00000203666	1	1696	245133549	Deletion	2
					245133623	Insertion	5
PNLIPRP3	1	ENSG00000203837	10	3	118231356	Deletion	2
					118215310	Insertion	1
					118231360	Insertion	2
LCN6	-1	ENSG00000204003	9	4	139639190	Insertion	1
					139639192	Insertion	1
AGAP6	1	ENSG00000204149	10	7	51748681	Deletion	1
					51769222	Deletion	14
					51768674	Deletion	2
MICA	1	ENSG00000204520	6	227	31380001	Deletion	1
					31380160	Deletion	1
					31380157	Deletion	4
					31380161	Insertion	11
					31380161	Insertion	2
					31380161	Insertion	8
AC006486.1	1	ENSG00000204957	19	3	42747179	Deletion	1
					42747163	Deletion	8
PCDHA4	1	ENSG00000204967	5	1140	140186984	Deletion	1
					140186988	Deletion	2
MUC19	1	ENSG00000205592	12	1421	40843506	Deletion	1
					40875134	Deletion	1
					40826435	Deletion	2
					40867032	Deletion	2
					40830450	Deletion	4
					40875574	Insertion	1
					40879206	Insertion	2
CYP2D7P	-1	ENSG00000205702	22	6	42538040	Deletion	1
					42540327	Deletion	1
					42538514	Insertion	1
C21orf49	1	ENSG00000205930	21	3	34169317	Insertion	1
					34160986	Insertion	7
HLA-A	1	ENSG00000206503	6	154	29912028	Deletion	1
					29910728	Insertion	1
					29912395	Insertion	2
OR5K3	1	ENSG00000206536	3	4	98110067	Deletion	4
					98110406	Insertion	1
CCDC183	1	ENSG00000213213	9	5	139694454	Insertion	1
					139694457	Insertion	1
					139694456	Insertion	2
SRA1	-1	ENSG00000213523	5	1640	139931629	Insertion	1
					139931628	Insertion	2
TTLL3	1	ENSG00000214021	3	22	9876877	Deletion	1
					9876871	Deletion	2
					9876874	Deletion	2
					9877088	Deletion	2
LINC00955	1	ENSG00000216560	4	1539	3589697	Deletion	1
					3589719	Deletion	1
					3590823	Deletion	2
					3590872	Deletion	2
ZNF812	-1	ENSG00000224689	19	16	9800968	Deletion	1
					9801440	Deletion	5
MROH5	-1	ENSG00000226807	8	2	142451265	Deletion	1
					142477563	Deletion	1
					142506588	Deletion	1
					142459777	Insertion	1
OR2T7	1	ENSG00000227152	1	166	248604929	Insertion	1
					248605354	Insertion	1
					248604559	Insertion	2

OR52I1	1	ENSG00000232268	11	13	4615776	Deletion	1
					4615773	Deletion	2
HLA-B	-1	ENSG00000234745	6	306	31324493	Deletion	1
					31324496	Deletion	1
					31324601	Insertion	1
					31324489	Insertion	3
AP001468.1	-1	ENSG00000235878	21	25	47612508	Insertion	2
					47612512	Insertion	2
OR5G3	-1	ENSG00000241356	11	2215	56587500	Deletion	1
					56587650	Insertion	1
PSG4	-1	ENSG00000243137	19	76	43708974	Deletion	1
					43708978	Deletion	1
FOXD1	-1	ENSG00000251493	5	4	72743876	Deletion	1
					72743299	Insertion	2
PRKDC	-1	ENSG00000253729	8	8	48866909	Deletion	1
					48805816	Insertion	1
STX16-NPEPL1	1	ENSG00000254995	20	19	57266239	Deletion	1
					57266263	Deletion	2
RP11-297N6.4	-1	ENSG00000255046	8	41	11659375	Deletion	1
					11659516	Deletion	1
OR13C5	-1	ENSG00000255800	9	263	107360768	Deletion	1
					107361178	Deletion	4
OR13C2	-1	ENSG00000257019	9	1352	107367664	Deletion	2
					107367392	Deletion	4
MUC22	1	ENSG00000261272	6	4	30995114	Deletion	1
					30995245	Deletion	1
					30996491	Deletion	1
					30996488	Deletion	2
TUBB8P7	1	ENSG00000261812	16	672	90161965	Deletion	1
					90161003	Insertion	2
GFY	1	ENSG00000261949	19	6	49931887	Deletion	4
					49931882	Insertion	2
LCN6	-1	ENSG00000267206	9	4	139639190	Insertion	1
					139639192	Insertion	1
AC092850.1	1	ENSG00000267819	12	2	131781582	Deletion	2
					131781120	Insertion	1
AC026740.1	1	ENSG00000268885	5	385	668757	Deletion	1
					668429	Deletion	2
					668654	Deletion	4
					668663	Deletion	4
					668574	Insertion	1
AC020952.1	1	ENSG00000268953	19	6	31640469	Insertion	8
					31640477	Insertion	8
					31640485	Insertion	8
AC002472.1	-1	ENSG00000269103	22	48	21360718	Deletion	2
					21360676	Insertion	1
AC020629.1	1	ENSG00000269367	12	55	42624063	Deletion	4
					42624068	Insertion	14
					42624063	Insertion	4
					42624091	Insertion	4
					42624077	Insertion	5
AL031663.2	1	ENSG00000269549	20	29	44075130	Deletion	1
					44075137	Insertion	1
AL589739.1	1	ENSG00000269753	1	1708	2258754	Deletion	10
					2258626	Deletion	2
					2258772	Deletion	2
					2258740	Deletion	4
					2258896	Deletion	8
					2258590	Insertion	2
					2258930	Insertion	2
					2258590	Insertion	4
					2258940	Insertion	7
					2258860	Insertion	8
RP11-156E8.1	-1	ENSG00000272195	1	1704	245134086	Deletion	1
					245133549	Deletion	2
					245133623	Insertion	5
RP11-216L13.17	1	ENSG00000272896	9	5	139694454	Insertion	1
					139694457	Insertion	1
					139694456	Insertion	2

OR5AL1	1	ENSG00000272987	11	43	56180826	Insertion	1
					56180636	Insertion	2
PPP1R9B	-1	ENSG00000108819	17	2504	48227384	Insertion	2
					48227403	Insertion	2
SON	1	ENSG00000159140	21	2504	34948696	Deletion	1
					34948684	Insertion	1
B3GNT6	1	ENSG00000198488	11	2504	76751541	Deletion	1
					76751584	Deletion	1
					76751603	Deletion	1
IGHV3-21	-1	ENSG00000211947	14	2	106691867	Deletion	1
					106691871	Insertion	1
SCARF2	-1	ENSG00000244486	22	2504	20779973	Insertion	1
					20780024	Insertion	1
					20780029	Insertion	1

Table S5. NFS indels that introduce premature stop codons (introduced premature stop codons are underlined)

Ensembl_ID	Gene	Strand	Chr.	Position	Type	ref_seq	variant_seq
ENSG00000204869	IGFL4	-1	19	46543527	Deletion	tGGCCCTGCTTCCag	tag
ENSG00000231171	LINC01098	1	4	178897086	Deletion	tCTAaa	taa
ENSG00000133063	CHIT1	-1	1	203186950	Insertion	tgg	tAGGGACTGGGCGGGGCCATGGTCTgg
ENSG00000168970	JMJD7-PLA2G4B	1	15	42140022	Insertion	-	GCAGTGCAGCGGAGGCGGCAGCGCAGGCCCCACTGATGGCCGGGGCCT
ENSG00000124074	ENKD1	-1	16	67699975	Insertion	-	TGA
ENSG00000268461	AC078899.2	-1	19	20047782	Insertion	gaa	gTTTTTTaa
ENSG00000142025	DMRTC2	1	19	42355761	Insertion	c	cTAGAAA
ENSG00000167766	ZNF83	-1	19	53117029	Insertion	tgt	tgATGt

* two cases with stop retained variant

ENSG00000140853	NLRC5	1	16	57095835	Insertion	taa	tAAGAAAAAAAGAaa*
ENSG00000064270	ATP2C2	1	16	84497336	Insertion	tag	tAGTGGAag*
